# Low-dose post-transplant cyclophosphamide with G-CSF/ATG based haploidentical protocol provides favorable outcomes for SAA patients

**DOI:** 10.3389/fimmu.2023.1173320

**Published:** 2023-05-10

**Authors:** Xiaodi Ma, Zhengli Xu, Tingting Han, Yuanyuan Zhang, Wei Han, Haixia Fu, Xiaohui Zhang, Fan Lin, Xiaojun Huang, Lanping Xu

**Affiliations:** ^1^ Peking University People’s Hospital, Peking University Institute of Hematology, National Clinical Research Center for Hematologic Disease, Beijing Key Laboratory of Hematopoietic Stem Cell Transplantation, Peking University, Beijing, China; ^2^ Peking-Tsinghua Center for Life Sciences, Academy for Advanced Interdisciplinary Studies, Peking University, Beijing, China

**Keywords:** post-transplant cyclophosphamide, GvHD prophylaxis, haploidentical hematopoietic stem cell transplant, severe aplastic anemia, bone marrow transplantation

## Abstract

Haploidentical hematopoietic stem cell transplantation (haplo-HSCT), as one of the life-saving treatments for severe aplastic anemia (SAA), is widely used because of its great donor availability. Over decades, granulocyte colony-stimulating factor (G-CSF)/antithymocyte globulin (ATG)-based protocol (the so-called Beijing Protocol) has achieved favorable engraftment and survival outcomes. In this study, we modified the conventional Beijing Protocol: the full-dose Cyclophosphamide (Cy) (200 mg/kg in total) was divided into 42.75 mg/kg Cy on day -5 to day -2 and Low dose post-transplant Cy (PTCy) (14.5 mg/kg on days +3 and +4), hoping to reduce the incidence of severe acute graft-versus-host disease (aGVHD) and to guarantee successful and stable engraftment. Here we retrospectively reported and analyzed the data of first 17 patients with SAA who had received haplo-HSCT using this novel regimen between August 2020 and August 2022. The median follow-up was 522 days (range, 138-859 days). No patient developed primary graft failure. Four (23.5%) patients developed grade II bladder toxicity, two (11.8%) patients developed grade II cardiotoxicity. All patients achieved neutrophil and platelet engraftment at median times of 12 days (range, 11–20 days) and14 days (range, 8-36 days). During our follow-up, no patients developed grade III-IV aGVHD. The cumulative incidence of grade II and grade I aGVHD at 100 days was 23.5% (95% CI, 6.8%-49.9%) and 47.1% (95% CI, 23.0%-72.2%). Three patients (17.6%) developed chronic GVHD of skin, mouth, and eyes and all of which were mild. All patients are alive by the end of the follow-up, with a failure-free survival of 100%, which was defined as survival without treatment failures, such as death, graft failure, or relapse rate. The rate of cytomegalovirus (CMV) reactivation was 82.4% (95% CI, 64.3%-100%). The rate of Epstein-Barr virus (EBV) reactivation was 17.6% (95% CI, 3.8%-43.4%). No CMV disease and post-transplantation lymphoproliferative disorder (PTLD) occurred among these patients. In conclusion, the encouraging results of prolonged survival outcomes and reduced incidence of GVHD suggest promising effect of this novel regimen in haplo-HSCT for patients with SAA. Larger-sample prospective clinical trials are needed to confirm the effectiveness of this regimen.

## 1 Introduction

Patients suffering from severe acquired aplastic anemia (SAA) are at a high risk of death without prompt and appropriate treatment (1, 2). Because of its great donor availability, haploidentical hematopoietic stem cell transplantation (haplo-HSCT) has drawn increasing interest as a curative option for SAA (3–5). However, it remains currently relegated to rather late in the therapeutic algorithm, owing to concerns of life-threatening morbidity and transplant-related mortality (TRM) (6, 7).

Graft failure (GF) and severe graft-versus-host disease (GVHD) are two primary obstacles to successful haplo-HSCT (8, 9). Over decades, granulocyte colony-stimulating factor (G-CSF)/antithymocyte globulin (ATG)-based protocol (the Beijing Protocol (10)) has achieved favorable engraftment and survival outcomes both as an upfront and salvage treatment in SAA patients, which is comparable to those of transplantation from matched donors (11, 12). In the Beijing Protocol, all patients received conditioning regimens of busulfan (Bu), Cy and ATG. The infused graft consisted of G-CSF-stimulated bone marrow (BM) and peripheral blood stem cells (PBSCs). All patients were treated with cyclosporine A (CsA), mycophenolate mofetil (MMF) and short-term methotrexate (MTX) for GVHD prophylaxis. Whereas the relatively high incidence of acute GVHD (aGVHD), 29-33% for grade II-IV and 9-10% for grade III-IV aGVHD, hinders this platform from the more universal application (11–13). Apart from the Beijing Protocol, high-dose post-transplant cyclophosphamide (HD-PTCy, typically 50 mg/kg on days +3 and +4) has been successfully used in haplo-HSCT for SAA, and an inspiring decrease in aGVHD has been consecutively reported (5, 14–18). The European Society for Blood and Bone Marrow Transplantation (EBMT) Severe Aplastic Anemia Working Party reported the outcomes of HD-PTCy-based haplo-HSCT in a multicenter study (19). The incidence of grade II-III aGVHD was 23%, and there was no gradeIV aGVHD. It is particularly worrying that only 67% of patients achieved primary engraftment by day +28. Therefore, it is reasonable to modify the current two transplant protocols to exploit their strength and avoid unfavorable conditions.

Our team has demonstrated that combining low-dose PTCy of 14.5 mg/kg on days +3 and +4 (LD-PTCy) with ATG significantly reduced GVHD without compromising the potency of graft function in hematologic malignancies (20, 21). But it is undetermined if a similar procedure is feasible for patients with SAA. In this study, we modified the conventional Beijing Protocol (the transplantation procedure of which has been described in previous studies (10, 11)): the full-dose Cy (200 mg/kg in total) was divided into 42.75 mg/kg Cy on day -5 to day -2 and LD-PTCy (14.5 mg/kg on days +3 and +4), hoping to reduce the incidence of severe aGVHD and to guarantee successful and stable engraftment. Here we retrospectively reported and analyzed the clinical outcomes of 17 patients who received this modified protocol.

## 2 Methods

### 2.1 Patients

**Figure 1:**
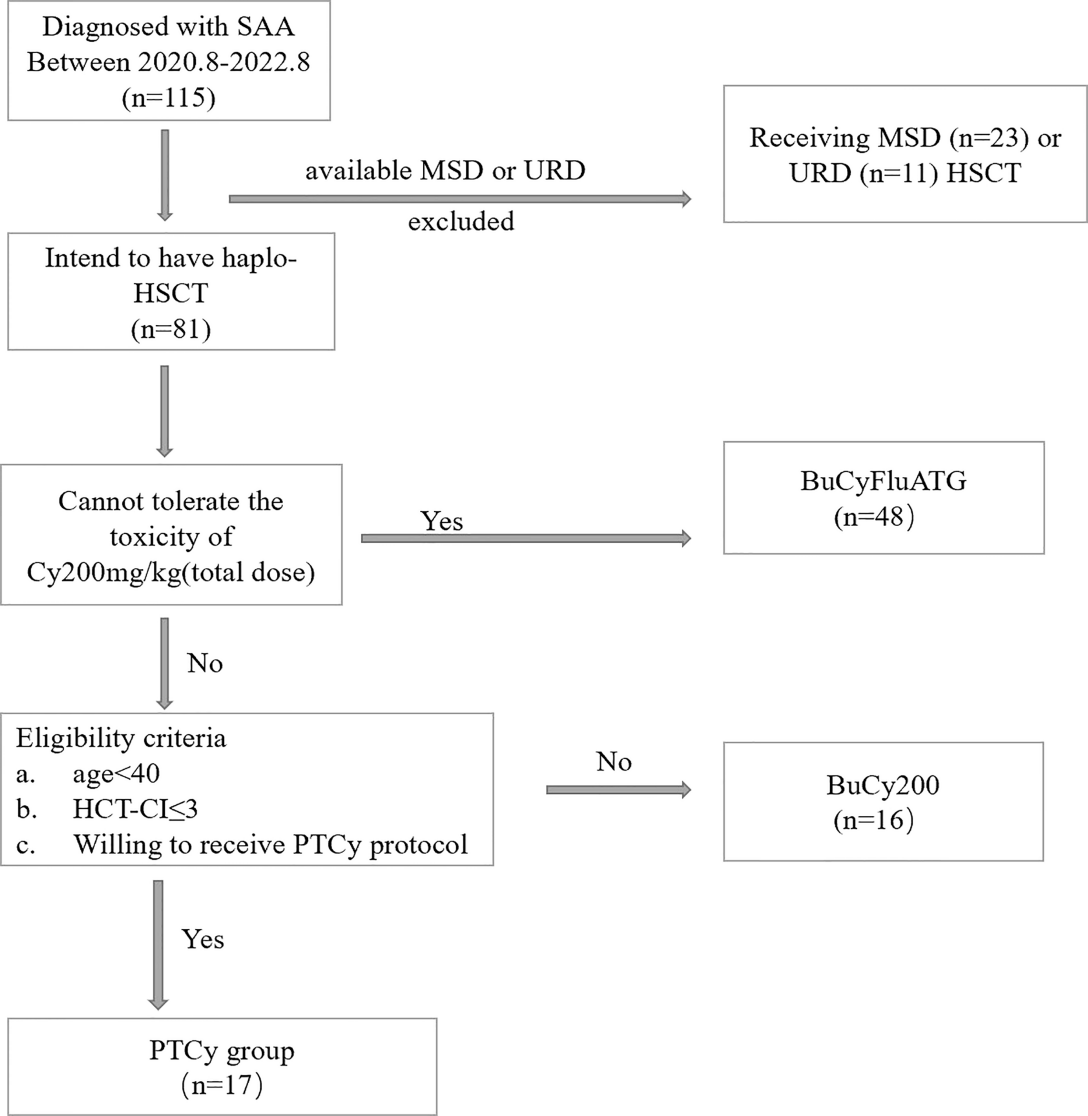
The Diagram of Patient Inclusion. BuCyFluATG regimen: Bu, 3.2mg/kg/d on days -8 and -7; Cy, 25 mg/kg/d on days -5 to -2; Flu, 30 mg/m^2^ daily on days -6 to -2, and ATG, 2.5 mg/kg daily on days -5 to -2; BuCy200 regimen: Bu, 3.2 mg/kg/d on days -8 and -7; Cy, 50 mg/kg/d, on days -5 to -2; ATG, 2.5 mg/kg/d, on days -5 to -2.

The effectiveness and safety of low-dose PTCy protocol for haplo-HSCT of hematological malignancies based on the Beijing protocol has been proved both in animal models and clinical trial in our center (20, 21). Starting from August 2020, we began to apply this protocol in haplo-HSCT for severe aplastic anemia. Between 1 August 2020 and 31 August 2022, 17 patients diagnosed with SAA accepting haplo-HSCT under the PTCy regimen at Peking University People’s Hospital Xizhimen Campus were enrolled in this study. The last follow-up date for all surviving patients was December 31, 2022 (**Figure 1**). This retrospective study summarized the data of the first 17 patients. Patients met the following criteria: (1) diagnosed with SAA or vSAA, as defined by the International Aplastic Anemia Study Group; (2) under 40 years old; HCT-CI ≤ 3; (3) can tolerate the toxicity of Cy 200mg/kg (total dose) and receive haplo-HSCT with the shift low-dose PTCy regimen; (4) no uncontrolled infectious diseases and liver, lung, renal and heart diseases; (5) lack of available HLA-identical, related sibling or unrelated donor. The protocol was approved by the ethics committee at Peking University People’s Hospital (PKUPH), and the study followed the Helsinki Declaration.

### 2.2 Definitions

Neutrophil engraftment was defined as the first of three consecutive days when the neutrophil count was ≥0.5 × 10^9^/L, while platelet engraftment was defined as the first occurrence of seven consecutive days with a platelet count ≥20 × 10^9^/L without transfusion. Patients with low donor chimerism who did not exhibit engraftment by day +28 were classified as having primary GF. Acute GVHD and cGVHD were diagnosed and graded according to international criteria (22, 23). Prognostic assessment included evaluation of overall survival (OS), failure-free survival (FFS), GVHD-free or failure-free survival (GFFS). OS was defined as the time from HSCT to death or the last follow-up for any reason. FFS was defined as survival without treatment failures, such as death, graft failure, or relapse. GFFS was defined as survival without grade III–IV aGVHD, moderate-to-severe cGVHD, or treatment failure including death, primary or secondary GF, and relapse. CMV viremia is defined as DNA monitoring greater than >1000 copies/ml. Regimen-related toxicity was defined and graded according to the Bearman criteria (24).

### 2.3 Transplantation procedures

**Figure 2:**
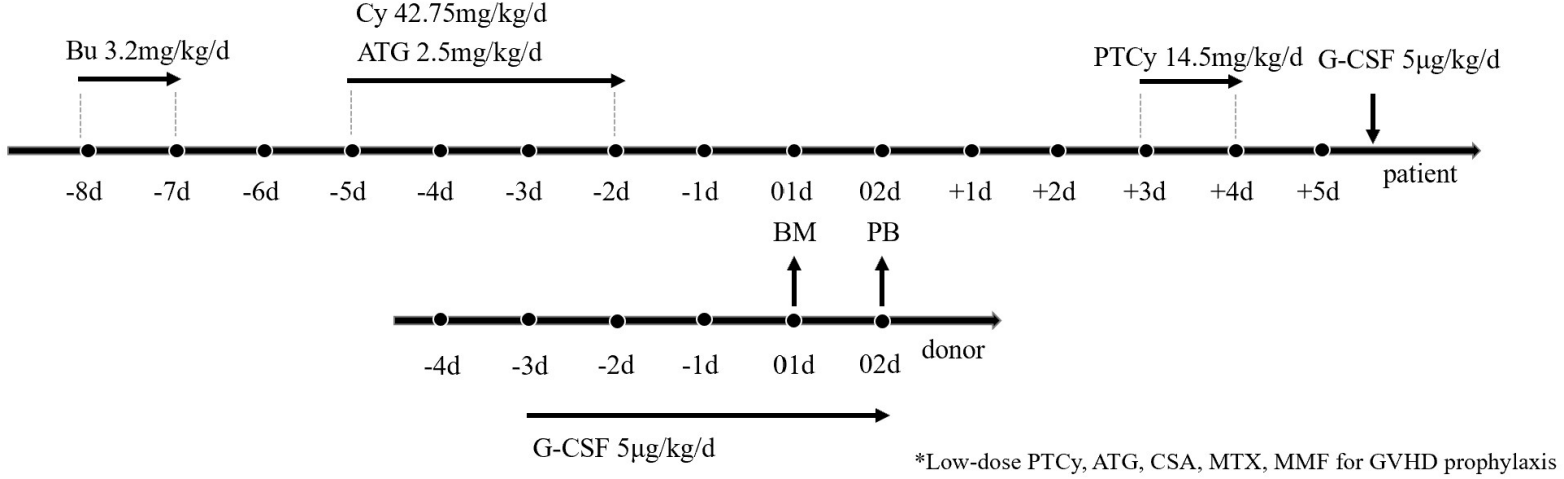
Transplantation Protocol of G-CSF/ATG/Low-dose PTCy regimen. Busulfan at 3.2 mg/kg was administered intravenously on days -7 to -6, followed by ATG at 2.5 mg/kg was administered intravenously on days -5 to -2, along with cyclophosphamide at 42.75 mg/kg intravenously on days -5 to -2. low-dose cyclophosphamide was administered from day 3 to day 4 at 14.5 mg/kg. For GvHD prophylaxis, aside from PTCy administered at 14.5mg/kg and ATG mentioned above, all patients received CsA, MMF, and short-term MTX. Donor stem cells were mobilized with subcutaneous G-CSF injection at a dosage of 5 μg/kg/day from day -3 until the last day of collection. Bone marrow (BM) grafts on day +1 and peripheral blood stem cells (PBSC) on day +2 were collected from donors.

The conditioning regimen, illustrated in **Figure 2**, consisted of four intravenous (i.v.) doses of 0.8 mg/kg busulfan (BU) on days -8 and -7, one intravenous dose of 42.75 mg/kg cyclophosphamide (CY) from days -5 to -2, and a four-day intravenous treatment of 2.5 mg/kg rabbit ATG (from SangStat) from day -5 to day -2. For GvHD prophylaxis, aside from PTCy which was administered at 14.5mg/kg and ATG mentioned above, all patients received CsA, MMF, and short-term MTX. CsA was administered intravenously (1.5mg/kg, q12h) from day -9 and switched to oral administration once bowel function returned to normal. MMF was given orally (0.5g q12h in adults or 0.25 g q12h in pediatric patients) from day -9, tapered in half on day +30, and discontinued on day +60. MTX was administered intravenously at a dose of 15mg/m^2^ on day +1, followed by doses of 10mg/m^2^ on days +3, +6, and +11.

BM grafts were collected on day 01, and peripheral blood stem cells (PBSC) were collected on day 02 from donors who received granulocyte colony-stimulating factor (G-CSF, 5μg/kg/d). BM cells were harvested to achieve a target volume of 10–12 mL/kg of donor weight or a target mononuclear cell (MNC) count of 2-4×10^8^/kg of recipient weight. On the fifth day of mobilization, PBSCs were collected with a COBE Blood Cell Separator (Spectra LRS; COBE BCT Inc., Lakewood, CO, USA) at a rate of 80 mL/min from a total blood volume of 10–12 L. The target MNC from BM and PB was 6-8×10^8^/kg of recipient weight. An additional collection of PBSCs was required the next day if the cell numbers from the previous 2 days were insufficient. Prophylactic therapy with ganciclovir (5mg/kg intravenously, twice daily, from days -8 to -2) was administered to all patients who underwent haploidentical transplantation. Preemptive therapy with ganciclovir (5mg/kg intravenously, twice daily) was started upon diagnosis of CMV viremia, and if the viremia became refractory, treatment was continued with a combination of foscarnet (60 mg/kg intravenously, twice daily) and immunoglobulin.

### 2.4 Statistical analysis

The main characteristics of patients were reported as descriptive statistics according to the available information. Continuous variables were presented as median and range. Survival analysis was conducted using the Kaplan-Meier method and long-rank test. For dichotomous variables, X² test and Fisher’s exact tests were used, while t-test was used for continuous variables. All p-values were two-tailed, and statistical significance was set at p<0.05. Statistical analysis was performed using SPSS 26.0 (SPSS, Inc., Chicago, IL, NY) and R 3.5.1 (http://www.r-project.org).

## 3 Results

### 3.1 Patients characteristics

A total of 17 patients received haplo-HSCT using the low-dose PTCy regimen and their characteristics are summarized in **Table 1**. The median follow-up was 522 days (range, 138-859 days). Seven patients were males (41.18%) and the median age of these patients was 12 (range, 5-39) years old. Two (11.76%) patients had no response to previous IST treatment, including ATG and CSA before HST; the remaining patients failed to respond to CsA ± stanozolol or other promoting-hematopoiesis drugs. The median age of the donors was 34 years (15 to 47 years), and 70.6% (12 of 17) of the donors were male. All donors were related to the patients, most commonly parents (58.8%). The blood types of 10 (58.82%) patients were matched between the donors and recipients, while those of 7 patients were mismatched.

**Table 1:** Patient Characteristics.

Patient	Age(yr)/sex	Donor Sex/relationship/age	HSCT year	Previous therapy	Severity	Interval Time*	HCT-CI	ECOG	ABO mismatch	HLA match	Median mononuclear cell (10^8^/kg)	Median CD34+ cells (10^6^/kg)	Median follow-up (months)
1	10/M	F/sister/17	2020.08	CSA	SAA	96	0	2	AB+ to AB+	8/10	14.71	3.46	28
2	11/F	M/father/34	2020.09	CSA	SAA	24	0	2	A+ to O+	6/10	14.05	3.58	27
3	16/M	F/sister/21	2020.10	CSA	SAA	42	0	2	A+ to A+	5/10	9.80	4.70	26
4	34/F	F/sister/37	2020.12	–	SAA	5	0	0	B+ to B+	5/10	15.03	4.45	25
5	8/F	M/father/33	2021.01	–	SAA	78	0	1	B+ to B+	5/10	11.81	3.71	23
6	19/M	M/father/47	2021.02	CSA	SAA	10	0	0	B+ to O+	5/10	7.82	4.14	23
7	12/F	M/father/42	2021.05	CSA	SAA	50	0	1	A+ to B+	7/10	12.91	3.81	19
8	18/M	M/father/43	2021.05	–	SAA with PNH	5	0	0	O+ to O+	5/10	14.59	3.73	19
9	11/F	M/father/52	2021.07	CSA	SAA	12	0	0	B+ to B+	5/10	10.45	3.38	14
10	11/M	F/mother/35	2021.11	CSA	SAA	46	0	1	A+ to O+	5/10	15.30	2.85	11
11	10/F	M/brother/15	2021.12	CSA	SAA	68	1	1	O+ to O+	5/10	14.50	6.47	9
12	14/M	M/father/36	2022.01	CSA	SAA	36	0	1	B+ to B+	6/10	11.11	3.12	9
13	5/F	M/father/33	2022.01	ATG CSA	vSAA (HAAA)	8	2	2	B+ to B+	5/10	11.34	4.83	9
14	7/M	M/father/32	2022.01	CSA	SAA	6	1	1	B+ to O+	5/10	14.33	6.08	8
15	27/F	M/brother20	2022.03	ALG CSA	vSAA	9	1	0	B+ to B+	6/10	13.90	6.53	6
16	25/F	M/brother20	2022.05	CSA	SAA	8	0	0	O+ to A+	7/10	11.61	5.27	4
17	39/F	F/sister/34	2022.08	CSA	SAA	2	0	0	B+ to A+	5/10	8.07	2.91	4

F, female; M, male; vSAA, very severe aplastic anemia; HAAA, hepatitis-associated-aplastic-anemia; PNH, paroxysmal nocturnal hemoglobinuria; CSA, cyclosporine A; ATG, antithymocyte globulin; HLA, human leukocyte antigen.

*interval time, interval time between diagnosis and HSCT.

### 3.2 Engraftment

**Figure 3:**
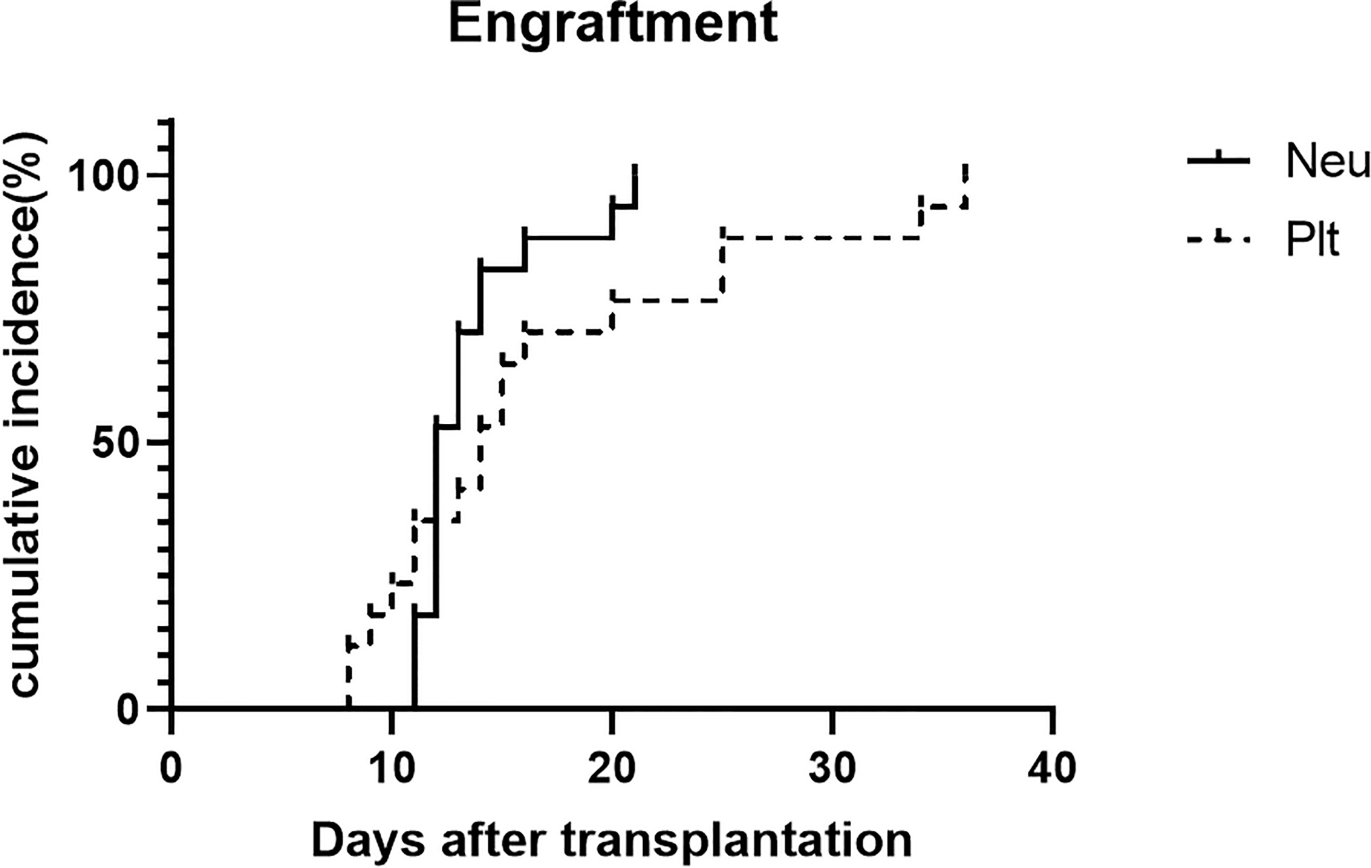
Engraftment of patients.

The median mononuclear cells infused in the HSCT were 12.91 (range, 7.82-15.3) ×10^8^/kg. The median CD34+cell count in grafts was 3.38×10^6^/kg (range, 0.46 to 6.53×10^6^/kg). Myeloid recovery and full donor chimerism were achieved in 17 patients after HSCT without primary graft failure. The median time for neutrophil engraftment was 12 days (range, 11–20 days). Platelet engraftment was achieved in all patients at median times of 14 days (range, 8-36 days). The incidence of engraftment is shown in **Figure 3**.

### 3.3 GVHD

**Figure 4:**
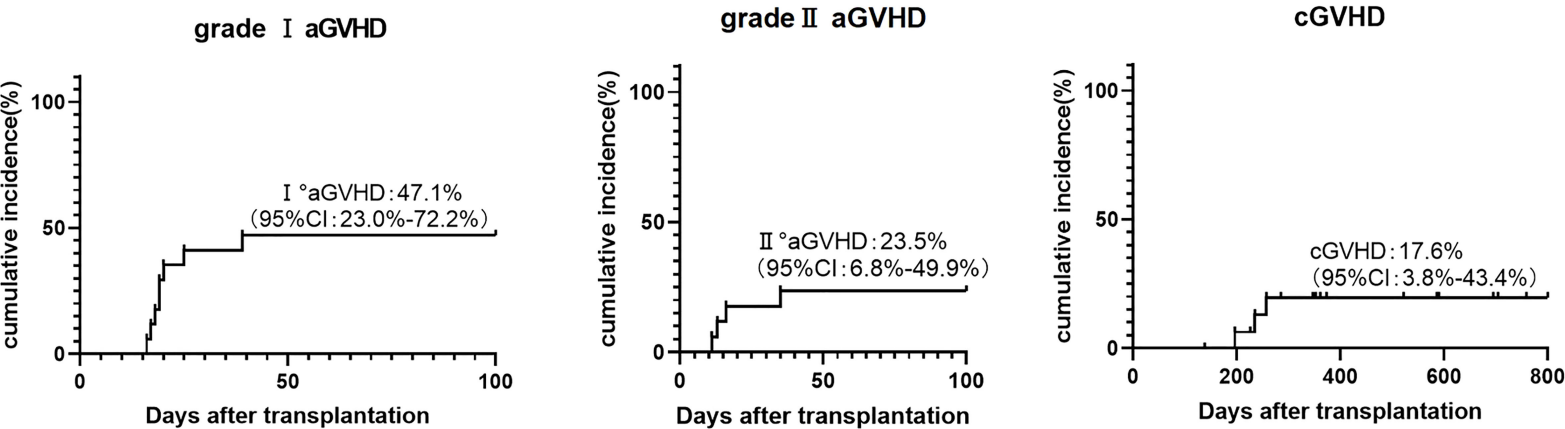
Cumulative incidence of grade I aGVHD, grade II aGVHD and cGVHD.

During our follow-up, no patients developed grade III-IV aGVHD. There were four cases of grade II aGVHD, including three cases of skin and gastrointestinal and one case of skin. The cumulative incidence of grade II and grade I aGVHD at 100 days was 23.5% (95% CI, 6.8%-49.9%) and 47.1% (95% CI, 23.0%-72.2%). Three patients (17.623.5%) developed chronic GVHD of skin, mouth, and eyes and all of which were mild (**Figure 4**). Two of them were diagnosed with skin-related cGVHD on the day+196 and day+235, respectively, presenting as flat mossy changes and skin sclerosis, which quickly recovered under methylprednisolone therapy. Another patient had symptoms in both mouth and eyes (**Table 2**).

**Table 2:** Patients outcomes.

Patient	Conditioning related toxicity Organ (grade)	Neu engraftment (range, days)	Plt engraftment (range, days)	CMV reactivation time	EBV reactivation time	Acute GVHD 1-4 Time/organ	Acute GVHD 2-4 Time/organ	Acute GVHD 3-4 Time/organ	Chronic GVHD Time/organ	Chimerism at last follow-up, %	Outcome
1	Bladder (II)	20d	25d	31d	38d	–	–	–	196d/skin	100% DC	Alive, transfusion independent
2		11d	8d	24d	27d	18d/skin (I)	–	–	235d/skin	100% DC	Alive, transfusion independent
3	Bladder (II)	11d	11d	43d	–	19d/skin (I)	–	–	–	100% DC	Alive, transfusion independent
4	Cardioid (II) Oral (I)	16d	25d	33d	–		16d/skin (I) and GI (I)	–	–	100% DC	Alive, transfusion independent Fibroblastoma of the pelvis
5	–	21d	8d	–	–	–	–	–	–	100% DC	Alive, transfusion independent
6	Bladder (II) GI (I)	12d	9d	48d	–		11d/skin (I) and GI (I)			100% DC	Alive, transfusion independent
7	CNS (II)	12d	13d	41d	–	20d/skin (I)		–	–	100% DC	Alive, transfusion independent
8	Bladder (I)	14d	15d	21d	–	–	–	–	–	100% DC	Alive, transfusion independent
9	–	13d	34d	35d	–		13d/skin (III)	–	–	100% DC	Alive, transfusion independent
10	Oral (I)	13d	16d	43d	–	25d/skin (I)	–	–	257d/mouth and eye	100% DC	Alive, transfusion independent
11	GI (I)	12d	15d	–	–	17d/skin (I)	–	–	–	100% DC	Alive, transfusion independent
12	Bladder (II)	12d	14d	23d	–		35d/skin (I) and GI (I)	–		100% DC	Alive, transfusion independent
13	–	12d	36d	27d	–	16d/skin (I)	–	–	–	100% DC	Alive, transfusion independent
14	–	13d	20d	28d	–	19d/skin (I)	–	–	–	100% DC	Alive, transfusion independent
15	Oral (I)	11d	10d	31d	–	39d/skin (I)	–	–	–	100% DC	Alive, transfusion independent
16	GI (I)	12d	11d	39d	45d	–	–	–	–	100% DC	Alive, transfusion independent
17	Cardioid (II)	14d	14d	–	–	–	–	–	–	100% DC	Alive, transfusion independent

Regimen-related toxicity was defined and graded according to the Bearman criteria.

GI indicates gastrointestinal; CNS, central nervous system; DC, donor chimerism; AIHA, autoimmune hemolytic anemia; CMV, cytomegalovirus viremia; GVHD, graft versus host disease.

### 3.4 Regimen-related toxicity and virus reactivation

All patients received the low-dose PTCy conditioning regimen as mentioned above (**Figure 1**). All of the regimen-related toxicity were mild or moderate. In all, 12 (71.1%) patients exhibited different degrees of toxicity (**Table 2**). Four (23.5%) patients developed grade II bladder toxicity, two (11.8%) patients developed grade II cardiotoxicity, and the other patients exhibited grade I-II regimen toxicity affecting organs like gastrointestinal tract, central nervous system, bladder, and oral. All toxicities happened are controlled during appropriate treatment and no death was caused by the conditioning regimen.

**Figure 5:**
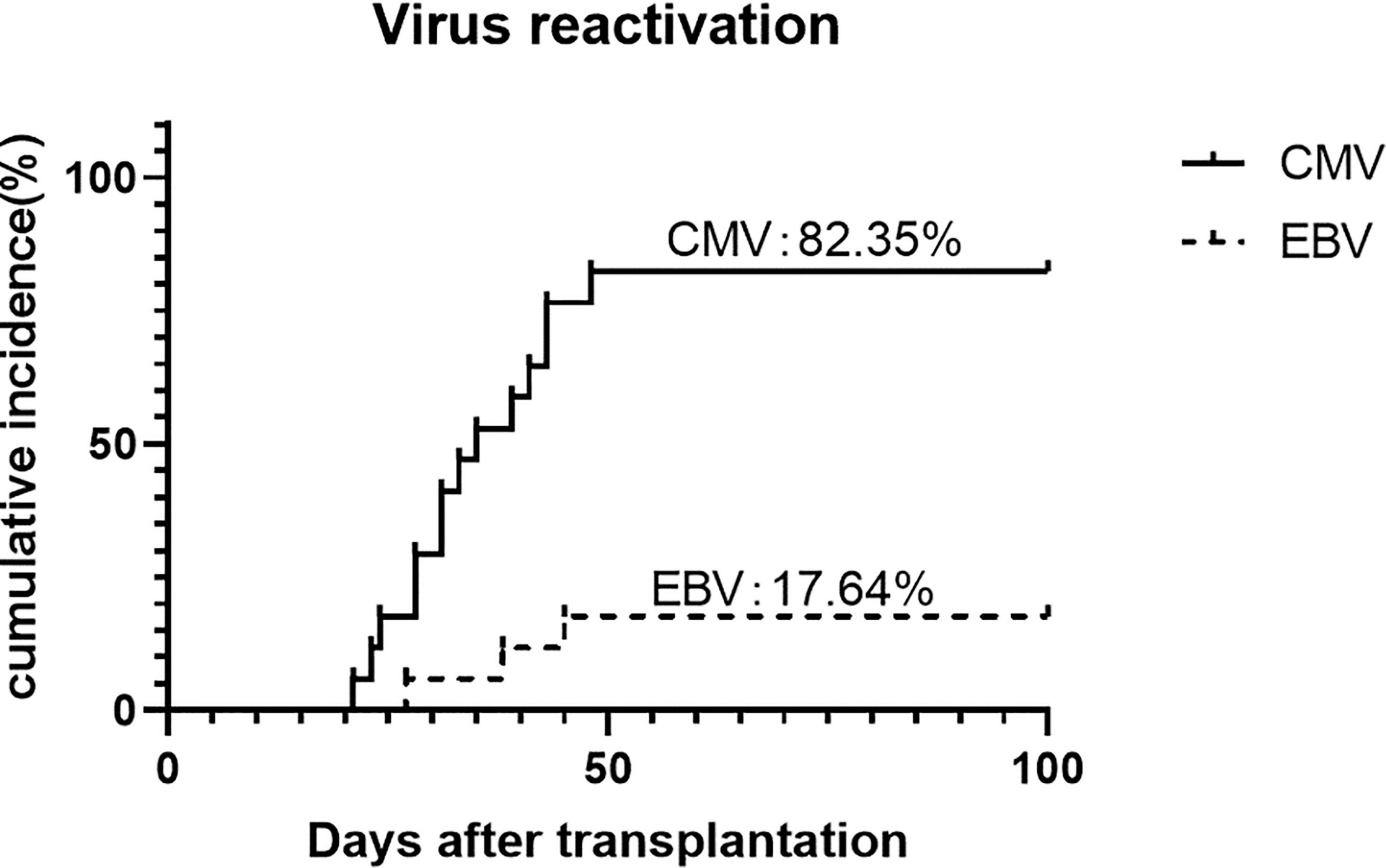
Cumulative incidence of CMV and EBV reactivation.

CMV reactivation was discovered in 82.4% (95% CI, 64.3%-100%) of the patients, with a median time of 35 days (range, 21-48 days). No CMV disease occurred during the follow-up. Three (17.6%) patients were discovered to have EBV reactivation and no post-transplantation lymphoproliferative disorder happened (**Figure 5**).

### 3.5 Survival and outcomes

The median follow-up was 522 days (range, 138-859 days). All patients survived and had no primary graft failure during our follow-up. The 2-year overall survival (OS) of these patients was 100%. Since there was neither death, graft failure, relapse, nor grade III-IV acute GVHD or moderate-to-severe chronic GVHD, the 2-year FFS and GRFS of these patients were also 100%. By the end of the follow-up, all patients were transfusion-independent at the end of the follow-up.

## 4 Discussion

The ideal conditioning regimen in SAA specifically should bring sustained engraftment, minimal regimen related toxicity, and a lack of GVHD. Both G-CSF/ATG-based and PTCy-based protocols have shown merits and demerits in haplo-HSCT for SAA (8, 15). Thus, our goal is to maximize the probability of engraftment and minimize GVHD. Based on a modified G-CSF/ATG/LD-PTCy protocol, our encouraging results of 100% primary engraftment, mild toxicity, reduced GVHD, and 100% probabilities of OS and GFFS have been achieved in haplo-HSCT for SAA patients.

During the early stage, GF is the primary concern after haplo-HSCT (25). With the Beijing protocol, the incidence of primary GF is less than 1-2% (11–13). In addition, fast and stable engraftment has been observed according to previous reports on G-CSF/ATG-based platforms (26). While the conventional HD-PTCy-based platforms usually include non-myeloablative regimens and de facto lead to a prolonged time to engraftment, as well as a higher risk of GF. Clay et al. (n = 8) and Esteves et al. (n = 16) have applied the HD-PTCy-based protocol with Flu 150 mg/m2, Cy 29 mg/kg, and total body irradiation (TBI) 2-6 Gy. Two primary GF (25%) and two secondary GF (12.5%), respectively, were documented in their studies (5, 14). The recent EBMT study analyzed multicentric data of thirty-three patients with SAA undergoing HD-PTCy-based haplo-HSCT from 2011 to 2017 (19). Notably, the incidence of myeloid engraftment is relatively low [67% (95% CI 51–83) at day +28 and 79% (95% CI 65–93) at day +100]. Therefore, it is reasonable to apply a more intensified conditioning when considering haplo-HSCT combining PTCy and G-CSF/ATG platform for SAA patients. More recently, Dezern et al. tried to add low-dose ATG (4.5 mg/kg) to the conventional HD-PTCy-based protocol in haplo-HSCT for SAA patients (16). A very concerning risk of GF (3 in an initial 7 patients) was noted in the early attempt, and they, therefore, increased the dose of TBI to 4Gy to ensure successful engraftment. Similar results have been revalidated in a prospective trial, in which 16% of patients developed GF and required a second transplant (27). On the other hand, several centers in China have administrated intravenous Bu with in-depth ablation of recipient-originated BM and hence sustained engraftment to replace TBI in conventional HD-PTCy-based protocol (28, 29). In the present study, we used a modified RIC regimen of Bu 3.2 mg/kg for 2 days and Cy 42.75 mg/kg for 4 days to shift the balance toward sustained engraftment. We demonstrated for the first time that the use of RIC regimen in the G-CSF/ATG/LD-PTCy platform enabled favorable engraftment with an inspiring short time to engraftment and no primary GF in haplo-HSCT for SAA patients. In line with our previous finding, no patient developed mixed chimerism and secondary GF (26, 30).

The other major problem for haploidentical transplantation in SAA patients is GVHD since there is no need for a graft-versus-tumor effect in SAA and it significantly contributes to TRM and inferior long-term survival. Huang et al. led two large multicenter studies and reported the incidences of grade II-IV aGVHD were 30.3% and 33.7%, grade III-IV aGVHD 7.9% and 10.1%, and cGVHD 22.4% and 30.6%, respectively, in SAA patients receiving upfront and salvage haplo-HSCT based on the G-CSF/ATG platform (11, 12). Of note, extended evidence reveals a very low rate of GVHD with the PTCy protocol (31, 32). The cumulative incidences were 12-26% for grade II-IV aGVHD and 0-28% for cGVHD, respectively (5, 28). In general, G-CSF/ATG can mitigate GVHD by eliminating alloreactive T cells and facilitating immune tolerance, while HD-PTCy can reduce alloreactive T cell proliferation, impair the function of surviving alloreactive T cells, and lead to preferential recovery of regulatory T cells (33, 34). Utilizing pre-transplant ATG and HD-PTCy, Dezern et al. have shown a rate of 11% for grade II-IV aGVHD and 8% for 2-year cGVHD. In addition, the rates of grade II-IV aGVHD and 1-year cGVHD were informed to be 16% and 26%, respectively, when giving the same protocol to patients with relapsed or refractory SAA (16). Recently, Wang and Chang et al. demonstrated that LD-PTCy can enhance the preventive effect of G-CSF/ATG on GVHD, and reduced incidences of aGVHD and cGVHD without compromising graft function and anti-tumor effect have been prospectively confirmed in a group of patients with high GVHD risk (21). Consistent with previous experience, we found 23.5% of patients developed grade II aGVHD and the rate of cGVHD was 17.6%. Inspiringly, no patients developed severe aGVHD or moderate-to-severe cGVHD in this small cohort. Our result again proves that LD-PTCy, in concert with ATG, is sufficient to induce immune tolerance. In this way it could further mitigates GVHD and paves the way for using G-CSF/ATG/LD-PTCy protocol in the SAA population.

In this study, we retained the standard dose of ATG from Beijing protocol to provide adequate immunosuppression. We excluded radiation from the regimen to avoid post-transplantation malignancies and growth impairment. Additionally, we shifted low-dose PTCy in the regimen to reduce incidence of GVHD and avoid delayed immune reconstruction. Consequently, the sustained engraftment and low incidences of GVHD have brought encouraging survival. Previously, the OS was 78-90% and GFFS was 60-70% for patients with SAA after haplo-HSCT. Of note, no TRM, treatment failure, or severe GVHD were documented in our study, and therefore the probabilities of OS and GFFS were 100%. Furthermore, with the well-controlled cGVHD and sustained transfusion independence, one can expect a satisfying quality of life following haplo-HSCT (35, 36).

With the intensive immunosuppression, we also paid close attention to the viral infection. We found that the incidence of CMV viremia was relatively high, which was 83.3% in our study. In contrast, it was 19-23% according to Dezern et al. utilizing ATG 4.5mg/kg and HD-PTCy (16). For the record, most of the Chinese population is born CMV seropositive and therefore at a high risk of post-transplant CMV reactivation. The rates of CMV reactivation were 52-80% and 75-82% in the G-CSF/ATG setting and the HD-PTCy setting, respectively (36–38). Li et al. reported a cumulative incidence of CMV reactivation of 53.54% when applying ATG 6 mg/kg plus HD-PTCy protocol, which is similar to the design of Dezern’s. Though the profile of immune reconstitution was absent, we assumed that adding LD-PTCy to the standard dose ATG of 10 mg/kg further delayed immune reconstitution and was partly responsible for increased CMV reactivation. Despite that the rate of CMV reactivation was high, one should note that no CMV disease or CMV-related death occurred. The use of novel agents such as letermovir as CMV prophylaxis may be beneficial in the G-CSF/ATG/LD-PTCy setting (39). In addition, it is possible to extend the modification by reducing ATG dose, given the rationale for ATG de-escalation presented in a prospective study including SAA patients. On the contrary, the rate of EBV reactivation in our study was acceptable compared to previous studies, and no EBV-associated PTLD occurred. Given that PTCy may deplete EBV-harboring lymphocytes, as is done by prophylactic rituximab, the result makes sense.

It is also essential to find this modified regimen was well tolerated. Cardiotoxicity and hemorrhagic cystitis associated with high-dose cyclophosphamide have been extensively reported (40, 41). In this study, no fatal cardiotoxicity or other RRT was observed, although high-dose cyclophosphamide (cumulative dose of 200/kg) was retained in the conditioning and many patients were heavily transfused before transplantation. The rate of hemorrhagic cystitis was also acceptable with no severe hemorrhagic cystitis occurring.

This study has some limitations. Initially, with the small sample size, it takes caution to interpret our results. Long-term follow-up is needed to confirm the encouraging transplant outcomes. As shown in the current cohort, however, G-CSF/ATG/LD-PTCy protocol did lower the incidence of GVHD without imperiling potent engraftment in haplo-HSCT for SAA patients, which can hopefully further improve the therapeutic effect and expand the donor pool. Thus, a prospective clinical trial is about to conduct. Moreover, information on immune reconstitution, as well as further modification such as ATG dose reduction to avoid transplant-related morbidity, are needed.

In conclusion, our data revealed that it is feasible and effective to use the G-CSF/ATG/LD-PTCy protocol in haplo-HSCT for SAA. With RIC preparative regimen and the use of LD-PTCy, the incidence of severe GVHD was reduced and successful and stable engraftment was achieved.

## Data availability statement

The original contributions presented in the study are included in the article. Further inquiries can be directed to the corresponding author.

## Ethics statement

The studies involving human participants were reviewed and approved by the protocol was approved by the ethics committee at Peking University People’s Hospital (PKUPH), and the study followed the Helsinki Declaration. Written informed consent to participate in this study was provided by the participants’ legal guardian/next of kin.

## Author contributions

LX and XH designed the study. The data analysis and manuscript development were led by XM and ZX. All authors contributed to the article and approved the submitted version. 
